# Supplementary material for: The Prognostic Utilities of Various Risk Factors for Laryngeal Squamous Cell Carcinoma: A Systematic Review and Meta-Analysis
Source: Medicina (Kaunas). 2023 Mar 2;59(3):497. doi: 10.3390/medicina59030497 (PMC10057849; doi:10.3390/medicina59030497)
Supplement: Supplementary file 1 [file medicina-59-00497-s001.zip › Table S1.pdf]

Table S1. The characteristics of the included studies.

Study	Design	Number	Age, median (range) or mean(SD), y	Sex (Male/Female)	Nation	Laryngeal tumor stage	Extracted outcomes
Nichols 2012	Cohort	75	NA	66/9	UK	T1/T2	Age, gender, T stage, smoking, alcohol
Dziegielewski 2012	Cohort	258	64.2 (36-92)	205/53	Canada	T3/T4a	Treatment modality
Dionysopoulos 2013	Cohort	289	63 (36-82)	277/12	Greece	T1/T2/T3/T4	Nodal involvment, tumor subsite
Zhang 2013	Cohort	205	61.8±10.6	197/8	China	T1/T2/T3/T4	Age, TMN stage, T stage, node involvement, tumor subsite, smoking, alcohol, Charlson score, patholic differentiation
Timmermans 2015	Cohort	166	61.9 (11.3)	124/42	Netherlands	T3/T4	Treatment modality, Age, sex, T stage, node involvement
Wong 2015	Cohort	140	66 (36-92)	121/19	UK	T1/T2/T3/T4	Sex, age, smoking, node involvement, TMN stage
Grover 2015	Cohort	969	59.2 (10.4)	774/195	USA	T4a	Age, TMN stage, node involvement, Charlson score, tumor subsite
Wang 2016	Cohort	120	60.6±8.6	118/2	China	T1/T2/T3/T4	Age, smoking, alcohol, tumor subsite, T stage, node involvement, TMN stage, pathologic differentiation
Tu 2015	Cohort	141	59 (36-87)	137/4	China	T1/T2/T3/T4	T stage, node involvement
Fu 2016	Cohort	420	60±9.1 (33-84)	413/7	China	T3/T4	Age, sex, smoking, alcohol, tumor subsite, T stage, node involvement, TMN stage, pathologic differentiation
Graboyes 2017	Cohort	1460	NA	531/143	USA	T3	Age, sex, Charlson score, tumor subsite, pathologic differentiation
Dyckhoff 2017	Cohort	769	61.9 (9.7)	626/58	Germany	T4	Treatment modality, age, node involvement, tumor subsite, Charlson score

Birkeland 2017	Cohort	244	NA	208/36	USA	T1/T2/T3/T4	T stage
Cheraghlo 2018	Cohort	726	NA	528/198	USA	T1/T2	Age, sex, Charlson score, tumor subsite, T stage, node involvement
Chen 2018	Cohort	361	60 (35-87)	353/8	China	T1/T2/T3/T4	Age, sex, tumor subsite, T stage, node involvement, TMN stage, pathologic differentiation
Oh 2019	Cohort	329	62 (57-66)	30/6	Canada	T4a	Treatment modality, age, alcohol, sex, node involvement
Patel 2019	Cohort	8703	NA	6601/2102	USA	T2/T3/T4	Age, sex, Charlson score, pathologic differentiation, T stage, node involvement, treatment modality
Zhou 2019	Cohort	232	63 (39-81)	192/40	China	T1/T2/T3/T4	Age sex, smoking, alcohol, tumor subsite, T stage, node involvement, TMN stage, pathologic differentiation
Song 2019	Cohort	137	NA	133/4	China	T1/T2/T3/T4	Tumor subsite, age, pathologic differentiation
Bates 2019	Cohort	11,237	NA	8472/3538	USA	T3/T4	Age, sex, Charlson score, T stage, node involvement, treatment modality
Boukovalas 2020	Cohort	362	64	294/68	USA	T1/T2/T3/T4	Age, sex, T stage, node involvement ,smoking, alcohol
Zhang 2020	Cohort	207	NA	198/9	China	T1/T2/T3/T4	Age, sex, alcohol, smoking, pathologic differentiation, TMN stage , T stage, node involvement
Lin 2021	Cohort	2094	NA	1712/382	China	T1/T2/T3/T4	Age, sex, pathologic differentiation, node involvement

CRT; chemoradiation therapy, TL; total laryngectomy, RT; radiation therapy, HR; Hazard ratio.
