# Supplementary material for: The Prognostic Utilities of Various Risk Factors for Laryngeal Squamous Cell Carcinoma: A Systematic Review and Meta-Analysis
Source: Medicina (Kaunas). 2023 Mar 2;59(3):497. doi: 10.3390/medicina59030497 (PMC10057849; doi:10.3390/medicina59030497)
Supplement: Supplementary file 1 [file medicina-59-00497-s001.zip › Table S2.pdf]

Table S2. The methodological quality of the included studies.

Study (year)	Selection[a]				Comparability[b]		Exposure[c]			The Newcastle-Ottawa Scale
	**1**	**2**	**3**	**4**	**5A**	**5B**	**6**	**7**	**8**	
Nichols 2012	Yes	Yes	Yes	Yes	No	No	Yes	Yes	Yes	**7**
Dziegielewski 2012	Yes	Yes	Yes	Yes	No	No	Yes	Yes	Yes	**7**
Dionysopoulos 2013	Yes	No	No	Yes	Yes	Yes	Yes	Yes	Yes	**7**
Zhang 2013	Yes	Yes	Yes	Yes	No	No	Yes	Yes	Yes	**7**
Timmermans 2015	Yes	Yes	Yes	Yes	No	No	Yes	Yes	Yes	**7**
Wong 2015	Yes	No	Yes	Yes	No	No	Yes	Yes	Yes	**6**
Grover 2015	Yes	No	No	Yes	Yes	Yes	Yes	Yes	Yes	**7**
Wang 2016	Yes	No	Yes	Yes	No	No	Yes	Yes	Yes	**6**
Tu 2015	Yes	No	No	Yes	Yes	Yes	Yes	Yes	Yes	**7**
Fu 2016	Yes	Yes	Yes	Yes	No	No	Yes	Yes	Yes	**7**
Graboyes 2017	Yes	Yes	Yes	Yes	No	No	Yes	Yes	Yes	**7**
Dyckhoff 2017	Yes	No	No	Yes	Yes	Yes	Yes	Yes	Yes	**7**
Birkeland 2017	Yes	Yes	Yes	Yes	No	No	Yes	Yes	No	**6**
Cheraghlo 2018	Yes	Yes	Yes	Yes	No	No	Yes	Yes	Yes	**7**
Chen 2018	Yes	No	No	Yes	Yes	Yes	Yes	Yes	Yes	**7**
Oh 2019	Yes	Yes	Yes	Yes	No	No	Yes	Yes	No	**6**
Patel 2019	Yes	Yes	Yes	Yes	No	No	Yes	Yes	Yes	**7**
Zhou 2019	Yes	Yes	Yes	Yes	No	No	Yes	Yes	Yes	**7**

Song 2019	Yes	Yes	Yes	Yes	No	No	Yes	Yes	Yes	**7**
Bates 2019	Yes	No	Yes	Yes	No	No	Yes	Yes	Yes	**6**
Boukovalas 2020	Yes	No	No	Yes	Yes	Yes	Yes	Yes	Yes	**7**
Zhang 2020	Yes	Yes	Yes	Yes	No	No	Yes	Yes	No	**6**
Lin 2021	Yes	Yes	Yes	Yes	No	No	Yes	Yes	Yes	**7**

A star rating system was used to indicate the quality of a study, with a maximum of nine stars. A study could be awarded a maximum of one star for each numbered item within the selection and exposure categories

a: Selection (4 items): adequacy of case definition; representativeness of the cases; selection of controls; and definition of controls.

b:. Comparability (1 item): comparability of cases and controls on the basis of the design or analysis.

c:. Exposure (3 items): ascertainment of exposure; same method of ascertainment for cases and controls; and non-response rate (same rate for both groups).
