# Supplementary figures and images for: The Prognostic Utilities of Various Risk Factors for Laryngeal Squamous Cell Carcinoma: A Systematic Review and Meta-Analysis
Source: Medicina (Kaunas). 2023 Mar 2;59(3):497. doi: 10.3390/medicina59030497 (PMC10057849; doi:10.3390/medicina59030497)

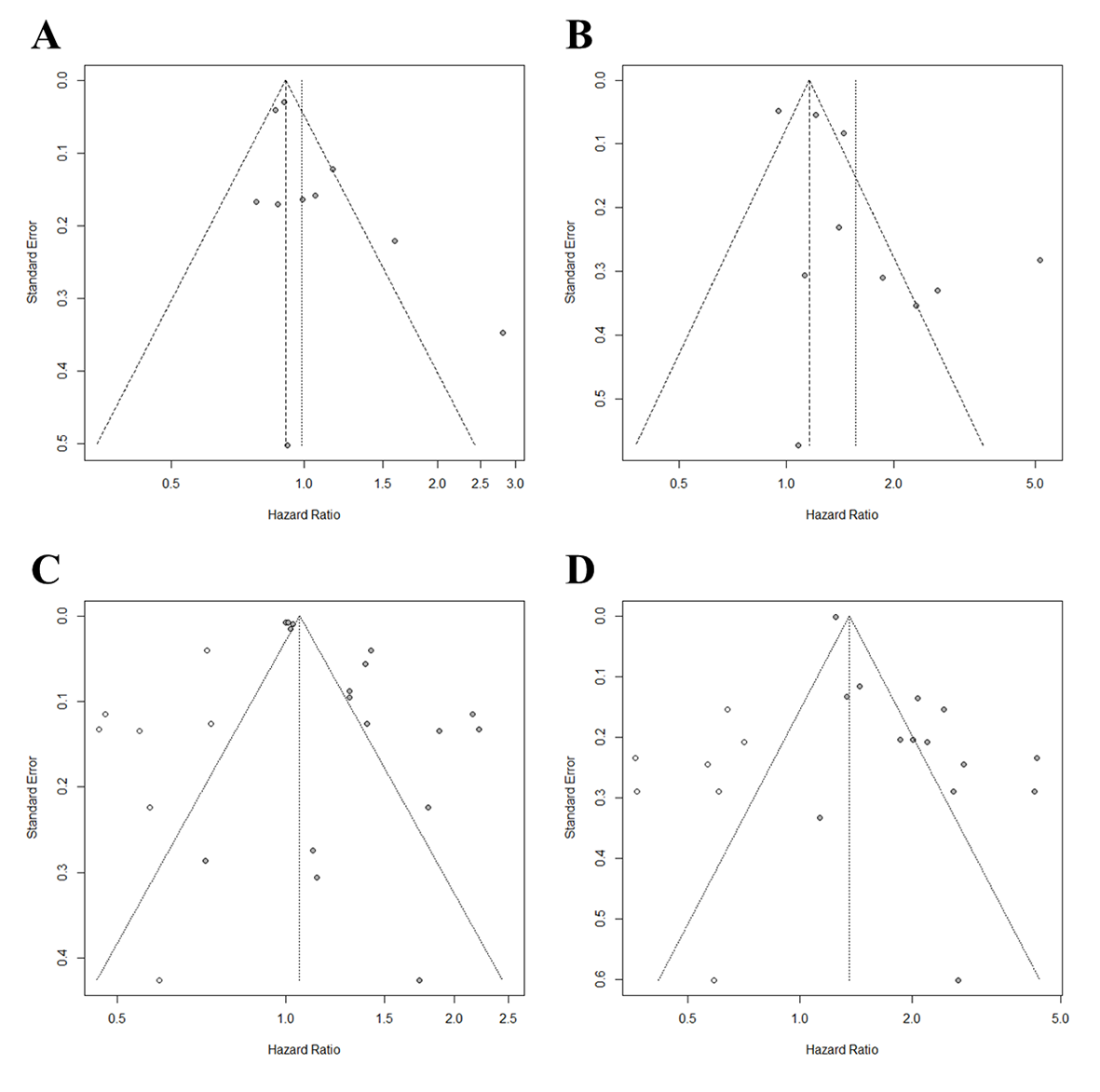

Supplement: Supplementary file 1 [file medicina-59-00497-s001.zip › Figure S1.tif]
